# A clinically compatible in vitro drug-screening platform identifies therapeutic vulnerabilities in primary cultures of brain metastases

**DOI:** 10.1007/s11060-024-04763-7

**Published:** 2024-07-10

**Authors:** Sebastian Jeising, Ann-Christin Nickel, Johanna Trübel, Jörg Felsberg, Daniel Picard, Gabriel Leprivier, Marietta Wolter, My Ky Huynh, Marlene B. Olivera, Kerstin Kaulich, Lena Häberle, Irene Esposito, Gunnar W. Klau, Julia Steinmann, Thomas Beez, Marion Rapp, Michael Sabel, Sascha Dietrich, Marc Remke, Jan F. Cornelius, Guido Reifenberger, Nan Qin

**Affiliations:** 1 Department of Neurosurgery, Medical Faculty, Heinrich Heine University, and University Hospital Düsseldorf, Düsseldorf, Germany; 2 Department of Pediatric Oncology, Hematology, and Clinical Immunology, Medical Faculty, Heinrich Heine University, University Hospital Düsseldorf, Düsseldorf, Germany; 3 Department of Hematology, Oncology, and Clinical Immunology, Medical Faculty, Heinrich Heine University, University Hospital Düsseldorf, Düsseldorf, Germany; 4 Spatial & Functional Screening Core Facility, Medical Faculty, Heinrich Heine University, Düsseldorf, Germany; 5 Institute of Neuropathology, Medical Faculty, Heinrich Heine University, and University Hospital Düsseldorf, Düsseldorf, Germany; 6 German Cancer Consortium (DKTK), partner site Essen/Düsseldorf, Düsseldorf, Germany; 7 Department of Computer Science, Heinrich Heine University Düsseldorf, Düsseldorf, Germany; 8 Institute of Pathology, Medical Faculty, Heinrich Heine University, University Hospital Düsseldorf, Düsseldorf, Germany; 9 Mildred Scheel School of Oncology Aachen Bonn Cologne Düsseldorf (MSSO ABCD), Düsseldorf, Germany; 10 Department of Pediatric Hematology and Oncology, University Medical Center of Saarland, Homburg/Saar, Germany

**Keywords:** Brain metastasis, Spheroid cultures, High-throughput drug screening, Personalized medicine

## Abstract

**Purpose:**

Brain metastases represent the most common intracranial tumors in adults and are associated with a poor prognosis. We used a personalized in vitro drug screening approach to characterize individual therapeutic vulnerabilities in brain metastases.

**Methods:**

Short-term cultures of cancer cells isolated from brain metastasis patients were molecularly characterized using next-generation sequencing and functionally evaluated using high-throughput in vitro drug screening to characterize pharmacological treatment sensitivities.

**Results:**

Next-generation sequencing identified matched genetic alterations in brain metastasis tissue samples and corresponding short-term cultures, suggesting that short-term cultures of brain metastases are suitable models for recapitulating the genetic profile of brain metastases that may determine their sensitivity to anti-cancer drugs. Employing a high-throughput in vitro drug screening platform, we successfully screened the cultures of five brain metastases for response to 267 anticancer compounds and related drug response to genetic data. Among others, we found that targeted treatment with JAK3, HER2, or FGFR3 inhibitors showed anti-cancer effects in individual brain metastasis cultures.

**Conclusion:**

Our preclinical study provides a proof-of-concept for combining molecular profiling with in vitro drug screening for predictive evaluation of therapeutic vulnerabilities in brain metastasis patients. This approach could advance the use of patient-derived cancer cells in clinical practice and might eventually facilitate decision-making for personalized drug treatment.

**Supplementary Information:**

The online version contains supplementary material available at 10.1007/s11060-024-04763-7.

## Introduction

Approximately 20–25% of patients with metastatic cancers, including those with carcinomas of the lung, breast, kidney, and colon, as well as cutaneous melanoma patients, develop brain metastases (BMs), with BMs being overall 10 times more frequent than primary brain tumors [1]. Despite advancements in the treatment of the respective primary tumors and metastases in other organs, the incidence of BMs is increasing [1]. Understanding of the biology and molecular pathomechanisms of BMs has greatly improved over the past years, and novel treatment options, including stereotactic radiosurgery, as well as molecularly targeted pharmacotherapy and immune checkpoint inhibition, have emerged [2–4]. Consequently, the prognosis of BM patients has improved, however, median overall survival times are still limited as indicated by the range from 2 to 21 months in the population-based Surveillance Epidemiology and End Results database [5].

Patients with BMs are a complex and heterogeneous population, making it challenging to develop general recommendations for specific treatments [6]. Furthermore, most patients have already received several treatments for their primary cancers, increasing the likelihood of resistance to multiple lines of therapy of BMs [7]. Therefore, predictive molecular testing and in vitro drug screening of BM tissue samples may broaden the personalized landscape of treatment options for improved management of BM patients.

In this study, we aimed to develop a pipeline for investigating in vitro drug sensitivity of tumor cells isolated from BMs using low-passage patient-derived tumor spheroid cultures and high-throughput drug screening. By integrating individual drug response results with tumor-associated genetic alterations, we identified potential personalized therapeutic options in individual BM patients. Hence, our approach bears the potential to be further developed towards personalized medicine for BM patients.

## Materials and methods

### Ethical approval and collection of patient samples

Ethical approval was obtained from the Ethics Committee of the Medical Faculty, Heinrich Heine University Düsseldorf (protocol No. 2020 − 1124). All patients gave their written informed consent for the use of their tissue samples and associated data for research purposes.

### Establishment of primary cell cultures from BMs

Primary tumor spheroid cultures were established from freshly resected surgical specimens, as previously described by Nolte et al. [8]. Briefly, neurosurgically resected sterile tumor tissue was cut into 1 mm diameter pieces and digested with 1X TrypLE (Gibco, Thermo Fischer Scientific, Waltham, USA) for 5 min at room temperature. The elimination of red blood cells was achieved using a red blood cell lysis buffer (Invitrogen, Carlsbad, USA) according to the manufacturer´s protocol. Tumor cells were cultured as 3D spheroids on Hema-coated low-attachment plates (Greiner Bio-One, Kremsmünster, Austria). The cells were grown in Dulbecco’s modified Eagle medium (Gibco) supplemented with 2% B27 (Gibco), 20 ng/ml bovine fibroblast growth factor (FGF, Peprotech, ThermoFisher Scientific), 20 ng/ml human epidermal growth factor (EGF, Peprotech), 5 µg/ml heparin (Sigma-Aldrich, St. Louis, USA), 1% Pen/Strep (Gibco) in standard culture conditions (humidified 37 °C, 5% CO_2_). Human fibroblast cells (HFB, NHDF-Ad) were obtained from Lonza (Basel, Switzerland) and were cultured using FGM-2 growth media.

### Blood processing

Blood samples were collected using BD Vacutainer K2E EDTA tubes (BD, Mississauga, Canada). To obtain peripheral blood cells (PBC) and total white cells, standard protocols were followed using EasySep RBC Depletion Reagent (Stemcell, Cologne, Germany). Specifically, immunomagnetic beads were used to remove red blood cells from fresh peripheral blood. Red blood cell-free PBCs were then cultured in Roswell Park Memorial Institute medium supplemented with 10% fetal bovine serum (Gibco).

### Immunohistochemistry

Formalin-fixed and paraffin-embedded tissue sections were deparaffinized in xylene and rehydrated over a graded ethanol series. To block endogenous peroxidase activity, sections were incubated in 3% hydrogen peroxide solution. The detection of HER2 protein expression was carried out using a polyclonal antibody against HER2 (A0485, Dako, Hamburg, Germany, diluted 1:1200). Anti-rabbit IgG was used as the secondary antibody. Horseradish peroxidase was used as a catalytic enzyme. Antibody binding was visualized with 3.3.-diaminobenzidine as a substrate for the horseradish peroxidase and chromogen. Finally, the slides were counterstained with hematoxylin and mounted for microscopic examination. Immunostaining for HER2 was evaluated according to the following immunoscore: 0, no positivity of the tumor cells; 1+, tumor cell clusters with weak or hardly perceptible membranous positivity; 2+, tumor cell clusters with weak to moderate complete, basolateral or lateral membranous positivity; 3+, tumor cell clusters with strong complete, basolateral or lateral membranous positivity.

### Gene-panel next-generation sequencing (NGS)

Tumor DNA was extracted from primary BM tissue samples and paired cell pellets from tumor spheroid cultures using the ReliaPrep gDNA Miniprep System (Promega, Mannheim, Germany) in accordance with the manufacturer’s protocol. Tumor tissue samples used for DNA extraction were histologically evaluated to show sufficient tumor cell content of 80% or more. Amplicon-based gene panel next-generation sequencing was performed as reported [9]. The NGS libraries were generated with DNA extracted from BM tissue samples and cell pellets using a customized gene panel for predictive molecular testing of non-small cell lung carcinoma (NSCLC) for BM from lung carcinomas or the commercially available Ion AmpliSeq^™^ Cancer Hotspot Panel v2 (Thermo Fisher Scientific, Waltham, USA) for BM from other primary tumors. The NSCLC panel covered mutational hot-spots in 27 cancer-associated genes (*ALK, BRAF, CTNNB1, EGFR, ERBB2, FGFR1, FGFR2, FGFR3, FGFR4, HRAS, IDH1, IDH2, KEAP1, KRAS, MAP2K1, MET, NRAS, NTRK1, NTRK2, NTRK3, PIK3CA, PTEN, RET, ROS1, STK11* and *TP53*), while the Ion AmpliSeq^™^ Cancer Hotspot Panel v2 covered mutational hot-spot regions in 50 cancer-associated genes (https://www.illumina.com/products/by-type/sequencing-kits/library-prep-kits/ampliseq-cancer-hotspot-panel.html). After sequencing, the amplicon sequences were aligned to the human reference genome GRCh37 (hg19) and the detected sequence variations were evaluated as reported [9].

### CellTiter-Glo luminescent cell viability (CTG) assay

The CellTiter-Glo reagent (Promega) was used to measure cell viability. The reagent was prepared according to the manufacturer’s instructions. The cell concentrations were validated to ensure logarithmic growth during the 72 h of incubation. The cells were seeded in 1536 well plates with Multidrop^™^ Combi Reagent dispenser (Thermo Fischer Scientific). After incubation, CellTiter-Glo was used to quench cells, and luminescence was measured using a Spark 10 M microplate reader (Tecan, Männedorf, Switzerland).

### Inhibitor libraries and drug screening

**Fig. 1:**
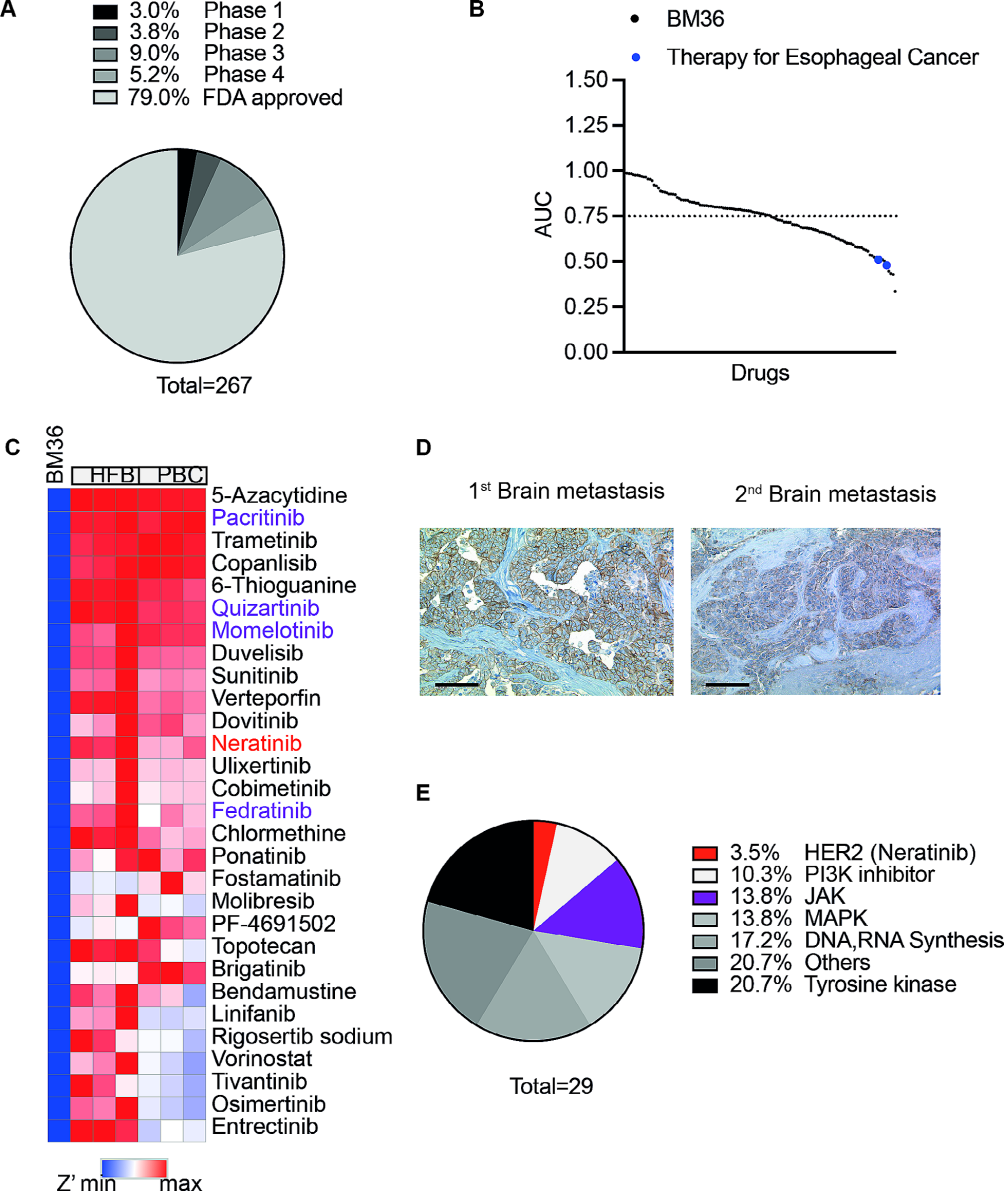
Combining molecular profiling within vitro drug screening to identify potential therapeutic options. **(A)** A graphical representation summarizing the different stages of clinical application and evaluation of the drugs included in the clinical library. **(B)** Plot of the fitted area under the dose-response curve (AUC) for 130 drugs with R2 > 0.8. The drugs have been sorted based on their AUC value. Blue dots: standard chemotherapy drugs used for esophageal cancer treatment. **(C)** A heatmap of Z’. Drugs with Z’<-2 were presented. **(D)** Immunohistochemical staining for HER2 expression in BM section of esophageal cancer in patient BM36. HER2-positive cells show a brown membranous stain. Left: Immunohistochemical staining of the first brain metastasis with intense complete membranous staining (HER2-Score 3+, scale bar: 100 μm); Right: Immunohistochemical staining of the relapsed brain metastasis with moderate staining on the basolateral and lateral sides of tumor cells (Score 2+, scale bar: 100 μm). **(E)** The distribution of selected potential drugs across target families

Drug screening was performed at the High-throughput Drug Screening Core Facility (HTS-CF) of the Medical Faculty at Heinrich Heine University Düsseldorf. Sample preparation and data processing were performed as described [10]. In brief, cancer cells were dissociated from the short-term spheroid cultures. In total, 2–3 million cells were required for examining drug response using a 1536-well format. A clinical library consisting of 267 anti-cancer compounds (TargetMol, Wellesley Hills, USA), including both FDA-approved medications and drugs undergoing clinical evaluation (Fig. 1A, Table S1). Each compound was tested across 6 to 8 different concentration levels, ranging from 0 to 10 µM [11]. The cellular response to the compounds was measured using a CTG assay and evaluated based on the normalized area under the dose-response curve (AUC).

### Statistical analysis

Drug response was evaluated by generating dose-response curves using non-linear regression in Python. The curves plotted log (concentration of inhibitor) versus response. To normalize the data, the cell viability at the lowest drug concentration was set to 100%. The parameters, including R2, AUC, and Z’, were calculated using an in-house results evaluation pipeline from HTS-CF.

## Results

### Establishing high-throughput drug screening using primary patient-derived cancer cells

In total, we tried to generate primary tumor spheroid cell cultures from BMs of 36 patients. The samples included various malignancies, including BMs from primary lung and breast carcinomas, cutaneous melanomas as well as other cancers (Table S2). The success rate in generating a short-term culture of cancer cells from BM tissue samples across all cancer types was 72% (26/36, Table S2). After 3–4 days, primary cancer cells formed dense aggregates, which were characteristically large, tightly packed spheroids of 100–800 μm in diameter (Fig. S1A).

In vitro cultivated cancer cells from the last six BM patients (BM28, BM31, BM32, BM33, BM35 and BM36) with sufficient tissue available for sequencing and for whom primary cultures were successfully established, were subjected to in vitro drug screening, thereby evaluating their response to 267 anti-cancer drugs. The median time between sample collection/start of in vitro cultivation and high-throughput drug screening was two weeks (range 1–4 weeks). 10 to 16 DMSO controls from each plate were plotted to calculate the coefficient of variation (CV) for evaluating variations in luminescence detection and errors in liquid handling (Fig. S1B). As proof of principle, on each plate, we employed panobinostat [12] and staurosporine [13], which have a wide range of toxic effects on different types of cancer cells. R2 was used to assess the goodness-of-fit for the response of the cells to these two drugs (Fig. S1C). Accurate screening results were selected based on the quality controls (CV < 15%, R2 > 0.8). Using these criteria, it was determined that the results obtained in five out of six BM primary cultures selected for in vitro drug testing met the quality standards.

Gene-panel next-generation sequencing was performed with tumor DNA extracted from tumor tissue samples and matched short-term cell cultures of these five patients. Identical genetic alterations were confirmed, with comparable mutant allele frequencies in both BM tissue and primary cultures (Table 1).

**Table 1:** Summary of genetic alterations detected in brain metastases and corresponding short-term cultures of five patients. The respective mutant allele frequencies are indicated in brackets

Sample ID	Primary tumor entity	DNA variants detected in BM tissue samples	DNA variants detected in BM-derived short-term cultures
BM28	NSCLC	*STK11* c.289_290 + 2delAAGT (AF = 59.6%) *TP53* c.734G > T:p.G245V (AF = 69.8%)	*STK11* c.289_290 + 2delAAGT (AF = 90.5%) *TP53* c.734G > T:p.G245V (AF = 92.5%)
BM31	Melanoma	*KIT* c.2447 A > T:p.D816V (AF = 29.5%) *JAK3* c.2164G > A:p.V722I (AF = 50.1%)	*KIT* c.2447 A > T:p.D816V (AF = 31.7%) *JAK3* c.2164G > A:p.V722I (AF = 51.0%)
BM32	GC	*ATM* c.2572T > C:p.F858L (AF = 40.8%)	*ATM* c.2572T > C:p.F858L (AF = 44.3%)
BM35	NSCLC	*FGFR3* c.1345 C > T:p.P449S (AF = 49.1%) *KRAS* c.34G > T:p.G12C (AF = 50.9%)	*FGFR3* c.1345 C > T:p.P449S (AF = 52.6%) *KRAS* c.34G > T:p.G12C (AF = 62.8%)
BM36	ESCA	*JAK3* c.2152G > C:p.V718L (1st BM: AF = 31.6%; 2nd BM: AF = 36.5%)	*JAK3* c.2152G > C:p.V718L (AF = 47.5%)

Abbreviations: NSCLC, non-small cell lung cancer; GC, gastric cancer; ESCA: esophageal carcinoma; AF, mutant allele frequency; BM, brain metastasis

### Using the pharmacogenomic approach to select potential personalized treatments

As stated above, we performed in vitro drug screening for a clinical anticancer library, in which nearly 80% of drugs are FDA-approved, and the remaining are in clinical evaluation (Fig. 1A). Using primary BM cancer cells from one patient (BM36) diagnosed with brain metastases derived from an adenocarcinoma of the esophagus exemplifies the capabilities of our drug screening platform. Dose-response curves were selected based on the goodness-of-fit parameter R2. In total, 130 drugs showed an R2 value above 0.8 in the BM-derived short-term cultures of this patient. To assess the potency and efficacy of each drug, we calculated the AUC and selected only those drugs with an AUC of at least 0.75 as effective compounds [14]. Using this criterion, out of 130 drugs, 60 drugs (46%) showed the expected effect on the patient-derived short-term cultures (Fig. 1B), including standard drugs used for clinical chemotherapy of esophageal cancers, such as epirubicin and doxorubicin [15, 16]. However, the dose-response curves of both drugs also showed high cytotoxicity toward peripheral blood cells (PBCs, Fig. S2).

To identify potential therapeutic options and exclude drugs with unwanted cytotoxic effects, Z’ was calculated [17]. Human fibroblasts (HFB) and PBCs were used as non-neoplastic control cells. Z’ lower than − 2 for primary cancer cells indicated a drug specifically targeting cancer cells but not the non-neoplastic control cells (Fig. 1C). Using this multiple criteria approach, 29 drugs (R2 > 0.8, AUC < 0.75, Z’ <-2) showed selective antitumor effects. As amplification and overexpression of the human epidermal growth factor receptor 2 (*HER2*) gene is an established therapy target in esophageal cancers, we performed immunohistochemical staining for HER2 expression on formalin-fixed and paraffin-embedded tissue sections, which indicated an overexpression in the two distinct brain metastases of patient BM36 (Fig. 1D). Nearly 25% of the drugs identified in the in vitro drug screen were targets of HER2-mediated signaling pathways, such as inhibitors for the mitogen-activated protein kinase (MAPK) pathway and phosphatidylinositol 3-kinase (PI3K) pathway [18]. Additionally, among six identified tyrosine kinase inhibitors, the novel oral pan-HER2 inhibitor, neratinib [19], was identified. Furthermore, gene panel NGS of BM36 tumor tissue and short-term cultures revealed a Janus kinase 3 (JAK3) somatic variant (V718L). In line, five of the 29 drugs (13.8%) showing in vitro efficacy were JAK inhibitors (Fig. 1E).

**Fig. 2:**
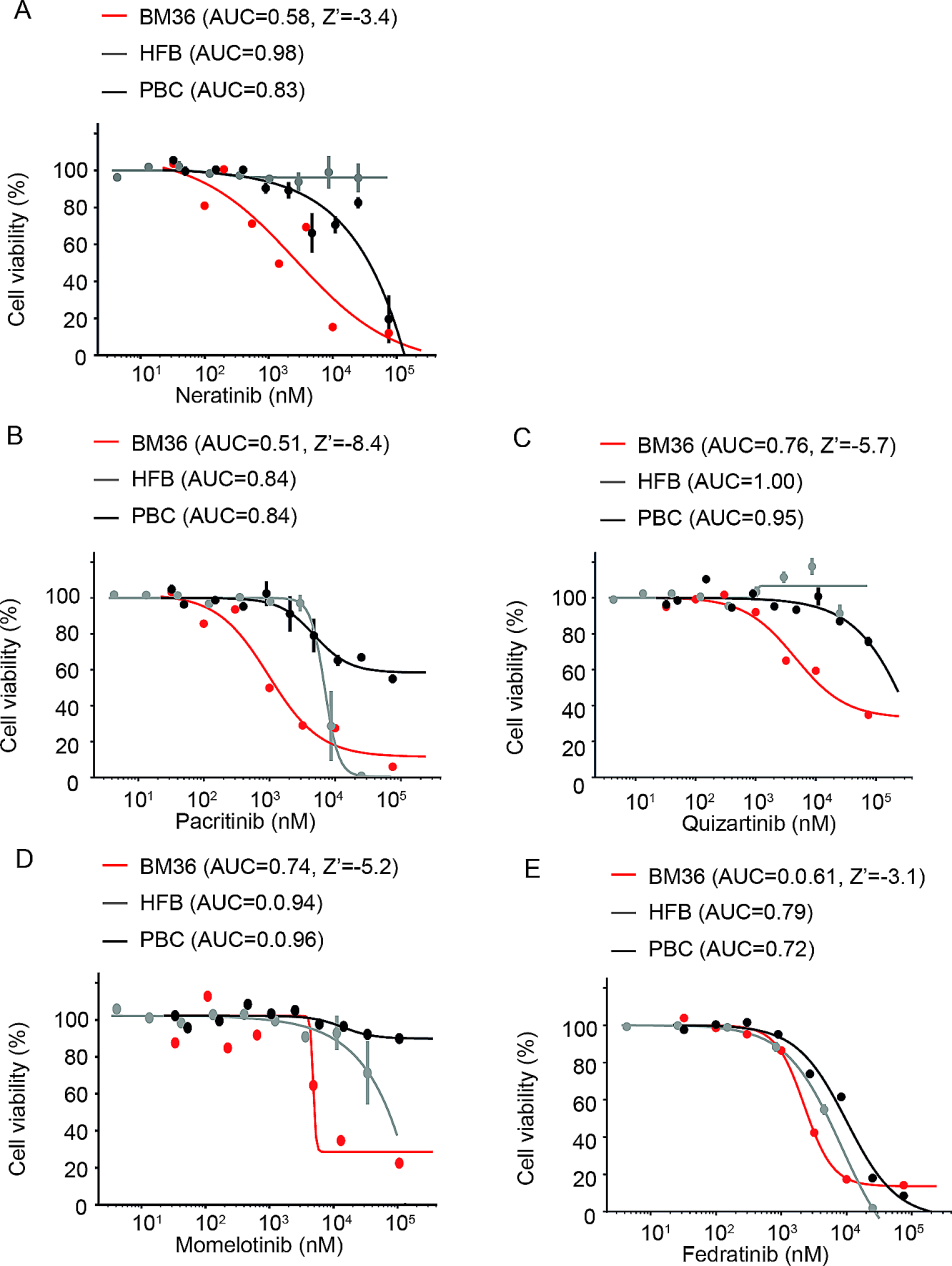
Dose-response curves of selected drugs in primary cultures of BM36. Short-term cultures of BM-derived tumor cells of patient BM36 showed lower AUC values for neratinib **(A)**, pacitinib **(B)**, quizartinib **(C)**, momelotinib **(D)**, and fedratinib **(E)** treatment as compared to human fibroblast (HFB) and peripheral blood cells (PBC). Cell viability was measured by CellTiterGlo assays

To further select targeted therapies based on molecular alterations, we graphed the dose-response curve for HER2 and JAK inhibitors. The graphs showed that the identified HER2 (Fig. 2A) and JAK (Fig. 2B-E) inhibitors significantly suppressed the growth of BM-derived tumor cells of patient BM36 while having little or no effect on HFB and PBCs.

Using the same strategy and cut-off criteria illustrated for case BM36, potential therapeutic options were identified for the four other BM patient primary cultures subjected to in vitro drug screening and gene panel NGS (Fig. S3). Taken together, we identified promising drugs that targeted either the specific genetic alterations, such as JAK3 inhibitors in BM31 and fibroblast growth factor receptor (FGFR) inhibitors in BM35, or drugs that targeted the signaling pathways downstream of the detected genetic alterations, such as extracellular-signal-regulated kinases (ERK), histone deacetylase (HDAC) and heat shock protein 90 (HSP90) inhibitors in BM28, and mitogen-activated protein kinase (MEK) inhibitors in BM32 (Table S3).

## Discussion

As the most frequent intracranial neoplasms in adults, BMs are commonly treated by neurosurgical resection or stereotactic radiosurgery, often followed by whole-brain radiation [3]. In addition, conventional chemotherapy, as well as molecularly guided targeted pharmacological treatments and/or immune checkpoint inhibition are increasingly used for treating BM patients [3]. While the combined multimodal treatment has improved the overall outcome of these patients, there still is a major clinical need to advance individualized treatment and thereby further prolong survival. Therefore, we investigated a potential personalized therapeutic approach for BM patients employing a translational preclinical platform for high-throughput in vitro drug screening combined with gene panel NGS-based mutational profiling of BM tissues and BM-derived short-term cultures.

In comparison to the use of patient-derived tumor xenograft models and preclinical in vivo drug screening [20–22], our approach based on primary cancer cell cultures is far less time-consuming and resource-demanding. We employed culturing cancer cells as tumor spheroids in serum-free medium as reported for in vitro cultivation of BM cells from primary lung cancer [8]. In comparison to cultivation in a serum-containing medium, the serum-free spheroid cultures used here are supposed to better preserve innate traits of the cancer cells, including their mutational profiles and phenotypic properties such as stem-like features that may impact drug response [23, 24]. To further preserve genetic and phenotypic features of the respective BM tissues, only shortly-term cultures of cancer cells that had undergone less than three splittings were investigated for selecting potentially effective drugs.

To employ drug screening of a more extensive collection of compounds with limited numbers of available cancer cells, we established a pipeline for use in a 1536-well format, which requires 90% less volume and fewer primary cells than a 384-well format [25]. For results evaluation, we used a comprehensive statistic package including the calculations of R2, AUC, and Z’, which effectively balances treatment effectiveness against toxicity and reliably determines potential hits. Moreover, this robust statistic package enabled us to select the potential hits using single patient drug response data, thereby expediting the translation of our findings into potential clinical applications.

**Fig. 3:**
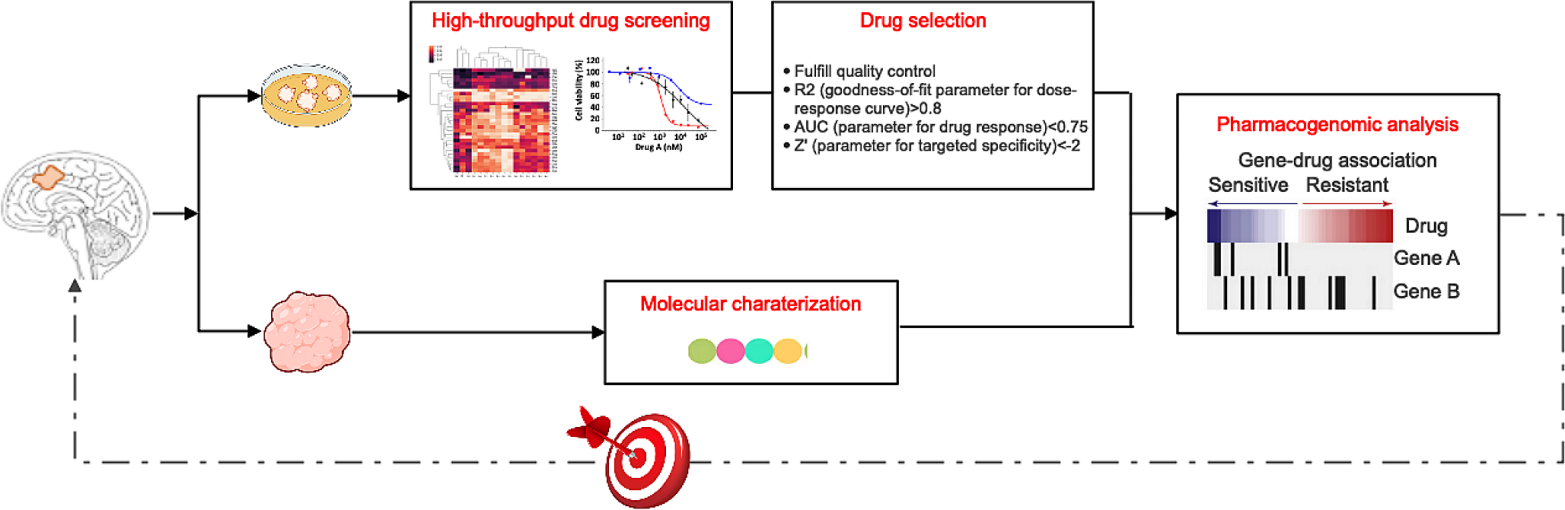
Schematic presentation of the combined molecular profiling and in vitro drug screening approaches used to identify potential personalized therapies in BM patients

Due to genetic heterogeneity and the associated divergence of BMs, we aimed to demonstrate the conceptual framework of our preclinical pipeline (Fig. 3) by presenting the results of an exemplary BM patient (BM36). Results from short-term cultured primary cancer cells-based high-throughput drug screening of BM36 revealed 60 drugs that strongly suppressed the growth of BM tumor cells of this patient, including standard chemotherapeutic drugs commonly used for treating esophageal cancer, namely, epirubicin and doxorubicin [26]. However, both drugs belong to the class of anthracyclines and exhibit severe cumulative toxicities, such as cardiotoxicity and secondary leukemia [27]. Compared to these conventional chemotherapies, targeted therapies are assumed to be more selective, i.e., show fewer side effects [28]. Patient BM36 harbored a HER2-positive BM. The primary tumor was treated with capecitabine and a HER2-directed agent (trastuzumab) [29], which showed improvements in the control of the systemic disease. However, large molecules like trastuzumab may not easily cross the blood-brain barrier [30], and this patient indeed developed BMs as the initial site of cancer relapse. In vitro drug screening of cancer cells derived from the HER2-positive BM of this patient identified a small molecule HER2 inhibitor, neratinib. In patients with HER2-positive metastatic breast cancer, neratinib has been shown to have intracranial activity [31] and treatment with this drug significantly reduced the risk of HER2-positive breast cancer progression and delayed the spread of this type of cancer to the brain [32]. Additionally, gene panel NGS revealed a *JAK3* missense variant in the first and second BM of this patient. JAK/STAT signaling is often altered in solid tumors and may drive tumor malignancy [33], suggesting that combination therapy with the identified HER2 and JAK inhibitors may be considered a potentially effective treatment.

The BM of patient BM28 carried a common variant in the serine/threonine kinase 11 gene (*STK11*) which is predictive for sensitivity to ERK inhibitors [34]. In our screening, we identified ulixertinib as the first-in-class ERK inhibitor, which has already shown promising results in treating low-grade glioma [35]. Additionally, BM28 carried a loss of function variant in *TP53* [36]. HDAC inhibitors or HSP90 inhibitors can degrade the mutant p53 protein [37]. A combined approach using ulixertinib (ERK inhibitor) with vorinostat (HDAC inhibitor) and tanespimycin (HSP90 inhibitor) [38, 39], which all have been reported to cross the blood-brain barrier, could potentially degrade the mutant p53 protein, and target STK11-mutant cells by abolishing S6 protein. BM31 carried a gain of function JAK3 variant [40]. The JAK3 inhibitor, pacritinib, which was also identified in the drug screening of BM36, could be a promising treatment in BM31 as well. In BM32, cancer cells with serine/threonine kinase (*ATM*) mutation could be specifically targeted by trametinib (MEK inhibitor) [41]. In addition, the *ATM* F858L mutation may induce the expression of *TP53* target genes [42], making this BM a suitable candidate for combined treatment with MEK, HDAC, and HSP90 inhibitors. The triple-angiokinase inhibitor nintedanib, which effectively blocks fibroblast growth factor receptor 1–3 (FGFR), has been investigated for lung diseases and could be considered a treatment option for BM35 [43].

Taken together, advanced next-generation sequencing techniques offer the potential to identify specific molecular targets and personalized drug treatment options for subsets but not all cancer patients. Our preclinical study provides proof of concept for using in vitro drug screening as orthogonal proof to support molecular recommendations and clinical decision-making. Moreover, in vitro drug screen may unravel therapy options in tumors without molecularly detectable druggable gene alterations. Other studies have reported on the establishment of patient-derived cell lines and organoid cultures from brain metastases [8, 44]. In addition, one study reported on the results of in vitro drug screening using an organoid cell culture established from a single case of brain metastasis from testicular carcinoma [45]. In comparison to other published studies that used comprehensive statistical analysis with the drug response of an extensive collection of primary cancer cells to select drugs [46–48], our study offers a robust testing strategy with the potential of clinical usability and demonstrates how to use drug responses in individual patient tumors to select potential hits.

We acknowledge that there are several shortcomings and the need for further improvements of our study. With a 72% success rate of in vitro primary tumor spheroid culture, our study falls within the upper range of reported success rates (9–78%) [49, 50]. However, this does not rule out the possibility of achieving even higher success rates when the entire pipeline from tissue resection, selection of viable tumor pieces, and in vitro cultivation is carried out by well-trained personnel following fully standardized and optimized procedures. To ensure that the primary culture recapitulates the intratumoral heterogeneity of corresponding tumors, we utilized an advanced serum-free culture condition, short culture periods, and a high-coverage gene panel NGS to analyze DNA extracted from primary cultures and corresponding brain metastases tissues. Our results demonstrate that the major tumor clones are preserved in the primary cultures. Nonetheless, we cannot rule out the possibility that minor subclones may escape from in vitro growth. To further optimize the experiment condition, comprehensive single-cell analyses would be required.

In addition, even though the blood-brain barrier is often compromised in malignant brain tumors, including brain metastases [51], certain drugs may still demonstrate low penetration to the BM tissue due to a blood-tumor barrier [52]. In case the in vitro screen identifies candidate drugs that are known to show limited penetration via the blood-brain barrier / blood-tumor barrier, application strategies involving local drug delivery [53] or ultrasound-mediated blood-brain barrier modulation [54], and nanoparticle technology [55] might be considered. Moreover, as the pattern and types of genetic alterations may vary between primary tumors and respective BM [56], combination treatments may be required to target both cancer sites.

Additional investigation into more advanced personalized in vitro models, such as patient-derived organoid cultures, co-cultures with non-neoplastic cells, and organs-on-a-chip platforms, could be warranted to further validate targeted anti-cancer effects versus unwanted side effects of selected candidate drugs under conditions that more closely recapitulate the multifaceted cellular and extracellular environment in BM tumors [57–59]. Eventually, newly designed clinical trials would be required to validate the clinical effectiveness of personalized approaches that integrate predictive molecular testing and in vitro drug screening in BM patients [60].

### Electronic supplementary material

Below is the link to the electronic supplementary material.

Supplementary Material 1

## Data Availability

No datasets were generated or analysed during the current study.
