# Supplementary material for: A clinically compatible in vitro drug-screening platform identifies therapeutic vulnerabilities in primary cultures of brain metastases
Source: J Neurooncol. 2024 Jul 10;169(3):613–23. doi: 10.1007/s11060-024-04763-7 (PMC11341655; doi:10.1007/s11060-024-04763-7)
Supplement: Supplementary file 1 — Supplementary Material 1 [file 11060_2024_4763_MOESM1_ESM.docx]

**Supplementary information**

**A clinically compatible *in vitro* drug-screening platform identifies therapeutic vulnerabilities in primary cultures of brain metastases**

**Table S1: Drugs in the investigated clinical drug library.**

| **Name** | **FDA/Clinical trial** | **CAS No.** | **M.Wt** | **Cat. No.** | **Company** |
| --- | --- | --- | --- | --- | --- |
| 17-AAG | FDA approved | 75747-14-7 | 585.69 | T6290 | TargetMOI |
| 5-Azacytidine | FDA approved | 320-67-2 | 244.2 | T1339 | TargetMOI |
| 5-Fluorouracil | FDA approved | 51-21-8 | 130.08 | T0984 | TargetMOI |
| 6-Mercaptopurine | FDA approved | 50-44-2 | 152.18 | T0010 | TargetMOI |
| 6-Thioguanine | FDA approved | 154-42-7 | 167.2 | T3089 | TargetMOI |
| Abarelix | FDA approved | 183552-38-7 | 1416.06 | T10217L | TargetMOI |
| ABC294640 | FDA approved | 915385-81-8 | 380.91 | T6750 | TargetMOI |
| Abiraterone | FDA approved | 154229-19-3 | 349.51 | T6216 | TargetMOI |
| ABL001 | FDA approved | 1492952-76-7 | 449.84 | T5177 | TargetMOI |
| ABT-199 | FDA approved | 1257044-40-8 | 868.44 | T2119 | TargetMOI |
| Acalabrutinib | FDA approved | 1420477-60-6 | 465.51 | T3626 | TargetMOI |
| Afatinib dimaleate | FDA approved | 850140-73-7 | 718.08 | T1773 | TargetMOI |
| AG-221 | FDA approved | 1446502-11-9 | 473.38 | T2346 | TargetMOI |
| Amifostine trihydrate | FDA approved | 112901-68-5 | 268.27 | T6381 | TargetMOI |
| Amonafide | FDA approved | 69408-81-7 | 283.33 | T6336 | TargetMOI |
| Amsacrine hydrochloride | FDA approved | 54301-15-4 | 429.92 | T5820 | TargetMOI |
| Anastrozole | FDA approved | 120511-73-1 | 293.37 | T0393 | TargetMOI |
| Bendamustine | FDA approved | 3543-75-7 | 394.72 | T0095 | TargetMOI |
| ARN-509 | FDA approved | 956104-40-8 | 477.43 | T2339 | TargetMOI |
| Artesunate | FDA approved | 88495-63-0 | 384.42 | T0433 | TargetMOI |
| Auranofin | FDA approved | 34031-32-8 | 678.48 | T1303 | TargetMOI |
| Avapritinib | FDA approved | 1703793-34-3 | 498.56 | T5109 | TargetMOI |
| Axitinib | FDA approved | 319460-85-0 | 386.47 | T1452 | TargetMOI |
| AZD-9291 | FDA approved | 1421373-66-1 | 595.71 | T3634 | TargetMOI |
| Bardoxolone methyl | FDA approved | 218600-53-4 | 505.69 | T6165 | TargetMOI |
| Baricitinib phosphate | FDA approved | 1187595-84-1 | 469.41 | T2360 | TargetMOI |
| BAY 80-6946 | FDA approved | 1032568-63-0 | 480.52 | T6322 | TargetMOI |
| Bazedoxifene acetate | FDA approved | 198481-33-3 | 530.67 | T2544 | TargetMOI |
| Belinostat | FDA approved | 866323-14-0 | 318.35 | T8517 | TargetMOI |
| Aprepitant | FDA approved | 170729-80-3 | 534.43 | T1743 | TargetMOI |
| Bestatin | FDA approved | 58970-76-6 | 308.37 | T1257 | TargetMOI |
| Bexarotene | FDA approved | 153559-49-0 | 348.49 | T6410 | TargetMOI |
| BIBF 1120 | FDA approved | 656247-17-5 | 539.62 | T1777 | TargetMOI |
| Bicalutamide | FDA approved | 90357-06-5 | 430.37 | T0380 | TargetMOI |
| Bleomycin sulfate | FDA approved | 9041-93-4 | 1512.62 | T6116 | TargetMOI |
| Talazoparib | FDA approved | 1207456-01-6 | 380.35 | T6253 | TargetMOI |
| Bortezomib | FDA approved | 179324-69-7 | 384.24 | T2399 | TargetMOI |
| Bosutinib | FDA approved | 380843-75-4 | 530.45 | T0152 | TargetMOI |
| Brigatinib | FDA approved | 1197953-54-0 | 584.09 | T3621 | TargetMOI |
| BSI-201 | FDA approved | 160003-66-7 | 292.03 | T6224 | TargetMOI |
| Busulfan | FDA approved | 55-98-1 | 246.3 | T0923 | TargetMOI |
| BYL-719 | FDA approved | 1217486-61-7 | 441.47 | T1921 | TargetMOI |
| Cabazitaxel | FDA approved | 183133-96-2 | 835.93 | T2543 | TargetMOI |
| Cabozantinib S-malate | FDA approved | 1140909-48-3 | 635.59 | T1797 | TargetMOI |
| CAL-101 | FDA approved | 870281-82-6 | 415.42 | T1894 | TargetMOI |
| Calcitonin（salmon） Acetate(47931-85-1(free base)) | FDA approved | TP1040L | 3431.85 | TP1040L | TargetMOI |
| Calcitriol | FDA approved | 32222-06-3 | 416.64 | T6316 | TargetMOI |
| Capecitabine | FDA approved | 154361-50-9 | 359.35 | T1408 | TargetMOI |
| Capsaicin | FDA approved | 404-86-4 | 305.41 | T1062 | TargetMOI |
| Captopril | FDA approved | 62571-86-2 | 217.29 | T1462 | TargetMOI |
| Carboplatin | FDA approved | 41575-94-4 | 371.25 | T1058 | TargetMOI |
| Carfilzomib | FDA approved | 868540-17-4 | 719.91 | T1795 | TargetMOI |
| Carmustine | FDA approved | 154-93-8 | 214.05 | T3091 | TargetMOI |
| Celecoxib | FDA approved | 169590-42-5 | 381.37 | T0466 | TargetMOI |
| CH5424802 | FDA approved | 1256580-46-7 | 482.62 | T1936 | TargetMOI |
| Chlorambucil | FDA approved | 305-03-3 | 304.22 | T0975 | TargetMOI |
| Chlormethine hydrochloride | FDA approved | 55-86-7 | 192.51 | T1417 | TargetMOI |
| CI-994 | FDA approved | 112522-64-2 | 269.3 | T1888 | TargetMOI |
| Ciclosporin A | FDA approved | 59865-13-3 | 1202.64 | T0945 | TargetMOI |
| Estramustine phosphate sodium | FDA approved | 52205-73-9 | 564.35 | T4451 | TargetMOI |
| Cladribine | FDA approved | 4291-63-8 | 285.69 | T2558 | TargetMOI |
| Clofarabine | FDA approved | 123318-82-1 | 303.68 | T0297 | TargetMOI |
| Cobimetinib | FDA approved | 934660-93-2 | 531.32 | T3623 | TargetMOI |
| Cortisone | FDA approved | 53-06-5 | 360.45 | T2884 | TargetMOI |
| Crizotinib | FDA approved | 877399-52-5 | 450.34 | T1661 | TargetMOI |
| CYT387 | FDA approved | 1056634-68-4 | 414.46 | T1849 | TargetMOI |
| Cytarabine | FDA approved | 147-94-4 | 243.22 | T1272 | TargetMOI |
| Dabrafenib mesylate | FDA approved | 1195768-06-9 | 615.67 | T8474 | TargetMOI |
| Dacarbazine | FDA approved | 4342-03-4 | 182.18 | T1120 | TargetMOI |
| Dacomitinib | FDA approved | 1110813-31-4 | 469.94 | T2483 | TargetMOI |
| Dapagliflozin | FDA approved | 461432-26-8 | 408.87 | T2389 | TargetMOI |
| Dasatinib | FDA approved | 302962-49-8 | 488.01 |  | TargetMOI |
| Daunorubicin hydrochloride | FDA approved | 23541-50-6 | 563.99 | T1511 | TargetMOI |
| Decitabine | FDA approved | 2353-33-5 | 228.21 | T1508 | TargetMOI |
| Deforolimus | FDA approved | 572924-54-0 | 990.21 | T6334 | TargetMOI |
| Dexamethasone | FDA approved | 50-02-2 | 392.46 | T1076 | TargetMOI |
| Dinaciclib | FDA approved | 779353-01-4 | 396.49 | T1912 | TargetMOI |
| Disulfiram | FDA approved | 97-77-8 | 296.54 | T0054 | TargetMOI |
| Docetaxel | FDA approved | 114977-28-5 | 807.88 | T1034 | TargetMOI |
| Dovitinib | FDA approved | 405169-16-6 | 392.43 | T6289 | TargetMOI |
| Doxorubicin hydrochloride | FDA approved | 25316-40-9 | 579.99 | T1020 | TargetMOI |
| Dutasteride | FDA approved | 164656-23-9 | 528.53 | T1499 | TargetMOI |
| Elesclomol | FDA approved | 488832-69-5 | 400.5 | T6170 | TargetMOI |
| Empagliflozin | FDA approved | 864070-44-0 | 450.91 | T1766 | TargetMOI |
| Entrectinib | FDA approved | 1108743-60-7 | 560.64 | T3678 | TargetMOI |
| Enzalutamide | FDA approved | 915087-33-1 | 464.44 | T6002 | TargetMOI |
| Enzastaurin | FDA approved | 170364-57-5 | 515.61 | T6280 | TargetMOI |
| Epirubicin hydrochloride | FDA approved | 56390-09-1 | 579.98 | T0125 | TargetMOI |
| EPZ-6438 | FDA approved | 1403254-99-8 | 572.74 | T1788 | TargetMOI |
| Erdafitinib | FDA approved | 1346242-81-6 | 446.55 | T3726 | TargetMOI |
| Eribulin mesylate | FDA approved | 441045-17-6 | 826 | A12804-0.5 | Hölzel |
| Erlotinib hydrochloride | FDA approved | 183321-74-6 | 393.44 | T0373 | TargetMOI |
| Cisplatin | FDA approved | 15663-27-1 | 300.05 | T1564 | TargetMOI |
| Etoposide | FDA approved | 33419-42-0 | 588.56 | T0132 | TargetMOI |
| Everolimus | FDA approved | 159351-69-6 | 958.22 | T1784 | TargetMOI |
| Exemestane | FDA approved | 107868-30-4 | 296.4 | T1587 | TargetMOI |
| Finasteride | FDA approved | 98319-26-7 | 372.55 | T0488 | TargetMOI |
| Floxuridine | FDA approved | 50-91-9 | 246.19 | T0964 | TargetMOI |
| Flutamide | FDA approved | 13311-84-7 | 276.21 | T0489 | TargetMOI |
| Fosbretabulin disodium | FDA approved | 168555-66-6 | 440.29 | T6272 | TargetMOI |
| Fulvestrant | FDA approved | 129453-61-8 | 606.77 | T2146 | TargetMOI |
| Ganetespib | FDA approved | 888216-25-9 | 364.4 | T2309 | TargetMOI |
| Gefitinib | FDA approved | 184475-35-2 | 446.91 | T1181 | TargetMOI |
| Gemcitabine | FDA approved | 95058-81-4 | 263.2 | T0251 | TargetMOI |
| Gilteritinib | FDA approved | 1254053-43-4 | 552.72 | T4409 | TargetMOI |
| Hexaminolevulinate hydrochloride | FDA approved | 140898-91-5 | 251.75 | T15477 | TargetMOI |
| Homoharringtonine | FDA approved | 26833-87-4 | 545.61 | T3380 | TargetMOI |
| Hydroxyurea | FDA approved | 127-07-1 | 76.06 | T0676 | TargetMOI |
| Idarubicin hydrochloride | FDA approved | 57852-57-0 | 533.95 | T6010 | TargetMOI |
| Imatinib mesylate | FDA approved | 220127-57-1 | 589.71 | T1621 | TargetMOI |
| Alvespimycin hydrochloride | Phase 1 | 467214-21-7 | 653.21 | T6297 | TargetMOI |
| Imiquimod | FDA approved | 99011-02-6 | 240.31 | T0134 | TargetMOI |
| Ingenol | FDA approved | 30220-46-3 | 348.43 | T5S2017 | TargetMOI |
| Duvelisib | FDA approved | 1201438-56-3 | 416.86 | T1988 | TargetMOI |
| Irinotecan | FDA approved | 97682-44-5 | 586.68 | T6228 | TargetMOI |
| Isoquercetin | FDA approved | 482-35-9 | 464.37 | T5S0754 | TargetMOI |
| Isotretinoin | FDA approved | 4759-48-2 | 300.44 | T1611 | TargetMOI |
| Itraconazole | FDA approved | 84625-61-6 | 705.63 | T1011 | TargetMOI |
| Zanubrutinib | FDA approved | 1691249-45-2 | 471.55 |  |  |
| Ixabepilone | FDA approved | 219989-84-1 | 506.7 | T6864 | TargetMOI |
| Lapatinib | FDA approved | 231277-92-2 | 581.06 | T0078 | TargetMOI |
| Larotrectinib sulfate | FDA approved | 1223405-08-0 | 526.51 | T6880 | TargetMOI |
| LDE225 | FDA approved | 956697-53-3 | 485.5 | T1926 | TargetMOI |
| LDK378 | FDA approved | 1032900-25-6 | 558.14 | T1791 | TargetMOI |
| LEE011 | FDA approved | 1211441-98-3 | 434.54 | T6199 | TargetMOI |
| Lenvatinib | FDA approved | 417716-92-8 | 426.85 | T0520 | TargetMOI |
| Letrozole | FDA approved | 112809-51-5 | 285.3 | T1590 | TargetMOI |
| LGX818 | FDA approved | 1269440-17-6 | 540.01 | T6487 | TargetMOI |
| Linsitinib | FDA approved | 867160-71-2 | 421.49 | T6017 | TargetMOI |
| Lomustine | FDA approved | 13010-47-4 | 233.7 | T1601 | TargetMOI |
| Lonafarnib | FDA approved | 193275-84-2 | 638.82 | T6302 | TargetMOI |
| Lurbinectedin | FDA approved | 497871-47-3 | 784.87 | HY-16293 | Hölzel |
| LY2835219 | FDA approved | 1231930-82-7 | 602.7 | T3111 | TargetMOI |
| Megestrol acetate | FDA approved | 595-33-5 | 384.51 | T1284 | TargetMOI |
| MEK162 | FDA approved | 606143-89-9 | 441.23 | T2508 | TargetMOI |
| Methotrexate | FDA approved | 59-05-2 | 454.44 | T1485 | TargetMOI |
| Mitomycin C | FDA approved | 50-07-7 | 334.37 | T6890 | TargetMOI |
| Mitotane | FDA approved | 53-19-0 | 320.04 | T1199 | TargetMOI |
| Mitoxantrone dihydrochloride | FDA approved | 70476-82-3 | 517.4 | T0158 | TargetMOI |
| MK-4827 tosylate | FDA approved | 1038915-73-9 | 492.59 | T6892 | TargetMOI |
| MLN9708 | FDA approved | 1239908-20-3 | 517.12 | T8397 | TargetMOI |
| Nelarabine | FDA approved | 121032-29-9 | 297.27 | T6603 | TargetMOI |
| Neratinib | FDA approved | 698387-09-6 | 557.04 | T2325 | TargetMOI |
| Nilotinib | FDA approved | 641571-10-0 | 529.52 | T1524 | TargetMOI |
| Nilutamide | FDA approved | 63612-50-0 | 317.22 | T0272 | TargetMOI |
| ODM-201 | FDA approved | 1297538-32-9 | 398.85 | T6915 | TargetMOI |
| Olaparib | FDA approved | 763113-22-0 | 434.46 | T3015 | TargetMOI |
| Oxaliplatin | FDA approved | 61825-94-3 | 397.29 | T0164 | TargetMOI |
| Paclitaxel | FDA approved | 33069-62-4 | 853.92 | T0968 | TargetMOI |
| Pacritinib | FDA approved | 937272-79-2 | 472.58 | T6020 | TargetMOI |
| Palbociclib | FDA approved | 571190-30-2 | 447.54 | T1785 | TargetMOI |
| Pamidronate Disodium | FDA approved | 110078-46-1 | 279.03 | T1776 | TargetMOI |
| Pazopanib hydrochloride | FDA approved | 635702-64-6 | 473.98 | T6930 | TargetMOI |
| PCI-32765 | FDA approved | 936563-96-1 | 440.5 | T1835 | TargetMOI |
| Pemetrexed | FDA approved | 357166-30-4 | 516.42 | T6226 | TargetMOI |
| Pemigatinib | FDA approved | 1513857-77-6 | 487.5 | T12401 | TargetMOI |
| Pentostatin | FDA approved | 53910-25-1 | 268.27 | T4006 | TargetMOI |
| Pexidartinib | FDA approved | 1029044-16-3 | 417.81 | T2115 | TargetMOI |
| PF-04449913 | FDA approved | 1095173-27-5 | 374.44 | T6514 | TargetMOI |
| PF-06463922 | FDA approved | 1454846-35-5 | 406.41 | T3061 | TargetMOI |
| Pipobroman | FDA approved | 54-91-1 | 356.06 | T4570 | TargetMOI |
| PKC412 | FDA approved | 120685-11-2 | 570.64 | T3211 | TargetMOI |
| Pomalidomide | FDA approved | 19171-19-8 | 273.24 | T2384 | TargetMOI |
| Ponatinib | FDA approved | 943319-70-8 | 532.56 | T2372 | TargetMOI |
| Pralatrexate | FDA approved | 146464-95-1 | 477.47 | T6120 | TargetMOI |
| Prednisone | FDA approved | 53-03-2 | 358.43 | T1018 | TargetMOI |
| Procarbazine hydrochloride | FDA approved | 366-70-1 | 257.76 | T1488 | TargetMOI |
| Regorafenib | FDA approved | 755037-03-7 | 482.82 | T1792 | TargetMOI |
| Relugolix | FDA approved | 737789-87-6 | 623.63 | T3630 | TargetMOI |
| Retinoic acid | FDA approved | 302-79-4 | 300.44 | T1051 | TargetMOI |
| Ritonavir | FDA approved | 155213-67-5 | 720.96 | T1525 | TargetMOI |
| Romidepsin | FDA approved | 128517-07-7 | 540.7 | T6006 | TargetMOI |
| Rucaparib | FDA approved | 459868-92-9 | 421.36 | T6127 | TargetMOI |
| Ruxolitinib phosphate | FDA approved | 1092939-17-7 | 404.36 | T3043 | TargetMOI |
| Selinexor | FDA approved | 1393477-72-9 | 443.31 | T6106 | TargetMOI |
| Selumetinib | FDA approved | 606143-52-6 | 457.68 | T6218 | TargetMOI |
| Sertraline | FDA approved | 79559-97-0 | 342.69 | T0482 | TargetMOI |
| Sorafenib tosylate | FDA approved | 475207-59-1 | 637.03 | T0093 | TargetMOI |
| Streptozocin | FDA approved | 18883-66-4 | 265.22 | T1507 | TargetMOI |
| Sunitinib | FDA approved | 557795-19-4 | 398.47 |  | TargetMOI |
| Tamoxifen | FDA approved | 10540-29-1 | 371.51 | T6906 | TargetMOI |
| Temozolomide | FDA approved | 85622-93-1 | 194.15 | T1178 | TargetMOI |
| Temsirolimus | FDA approved | 162635-04-3 | 1030.29 | T2145 | TargetMOI |
| Teniposide | FDA approved | 29767-20-2 | 656.66 | T1523 | TargetMOI |
| TG-101348 | FDA approved | 936091-26-8 | 524.68 | T1995 | TargetMOI |
| Thalidomide | FDA approved | 50-35-1 | 258.23 | T0213 | TargetMOI |
| Thio-TEPA | FDA approved | 52-24-4 | 189.22 | T1250 | TargetMOI |
| Thioridazine hydrochloride | FDA approved | 130-61-0 | 407.04 | T0365 | TargetMOI |
| Tipiracil hydrochloride | FDA approved | 183204-72-0 | 279.12 | T2366 | TargetMOI |
| Tivozanib | FDA approved | 475108-18-0 | 454.86 | T2456 | TargetMOI |
| Tofacitinib citrate | FDA approved | 540737-29-9 | 504.49 | T2398 | TargetMOI |
| Topotecan hydrochloride | FDA approved | 119413-54-6 | 457.92 | T1174 | TargetMOI |
| Toremifene citrate | FDA approved | 89778-27-8 | 598.08 | T1464 | TargetMOI |
| Trabectedin | FDA approved | 114899-77-3 | 761.84 | BV-B3084-250 | Enzo Life Sciences |
| Trametinib | FDA approved | 871700-17-3 | 615.39 | T2125 | TargetMOI |
| Trifluorothymidine | FDA approved | 70-00-8 | 296.2 | T1428 | TargetMOI |
| Triptorelin acetate(57773-63-4 free base) | FDA approved | 140194-24-7 | 1371.53 | T21410 | TargetMOI |
| Troglitazone | FDA approved | 97322-87-7 | 441.54 | T3170 | TargetMOI |
| Valproic acid sodium salt | FDA approved | 1069-66-5 | 166.2 | T1602 | TargetMOI |
| Valrubicin | FDA approved | 56124-62-0 | 723.64 | T7604 | TargetMOI |
| Vandetanib | FDA approved | 443913-73-3 | 475.31 | T1656 | TargetMOI |
| Vemurafenib | FDA approved | 918504-65-1 | 489.92 | T2382 | TargetMOI |
| Verteporfin | FDA approved | 129497-78-5 | 718.79 | T3112 | TargetMOI |
| Vinblastine sulfate | FDA approved | 143-67-9 | 909.06 | T1668 | TargetMOI |
| Vincristine sulfate | FDA approved | 2068-78-2 | 923.04 | T1270 | TargetMOI |
| Vindesine sulfate | FDA approved | 59917-39-4 | 852 | T22455 | TargetMOI |
| Vinflunine (ditartrate) | FDA approved | 194468-36-5 | 1117.10 | HY-B0628B | MedChem |
| Vinorelbine | FDA approved | 71486-22-1 | 778.93 | T0190 | TargetMOI |
| Vismodegib | FDA approved | 879085-55-9 | 421.3 | T2590 | TargetMOI |
| Volasertib | FDA approved | 755038-65-4 | 618.81 | T6019 | TargetMOI |
| Vorinostat | FDA approved | 149647-78-9 | 264.32 | T1583 | TargetMOI |
| VX-661 | FDA approved | 1152311-62-0 | 520.5 | T2263 | TargetMOI |
| Zoledronic acid | FDA approved | 118072-93-8 | 272.09 |  | TargetMOI |
| Cinobufotalin | Launched | 1108-68-5 | 458.54 | T4A2399 | TargetMOI |
| Delamanid | Launched | 681492-22-8 | 534.49 | T4202 | TargetMOI |
| Gimeracil | Launched | 103766-25-2 | 145.54 | T0987 | TargetMOI |
| Gossypol acetic acid | Launched | 12542-36-8 | 578.61 | T2730 | TargetMOI |
| Mozavaptan | Launched | 137975-06-5 | 427.54 | T6288 | TargetMOI |
| Noscapine | Launched | 128-62-1 | 413.42 | T7952 | TargetMOI |
| Pixantrone dimaleate | Launched | 144675-97-8 | 557.21 | T2394 | TargetMOI |
| R788 disodium hexahydrate | Launched | 914295-16-2 | 732.51 |  | V4 |
| Raltitrexed | Launched | 112887-68-0 | 458.49 | T6632 | TargetMOI |
| TAS-102 | Launched | 733030-01-8 | 871.53 | T3658 | TargetMOI |
| VAL-083 | Launched | 23261-20-3 | 146.14 | T17212 | TargetMOI |
| Pamiparib | NMPA approved | 1446261-44-4 | 298.31 | T5058 | TargetMOI |
| (-)-Epigallocatechin Gallate | Phase 4 | 989-51-5 | 458.38 | T2988 | TargetMOI |
| Andrographolide | Phase 4 | 5508-58-7 | 350.44 | T2898 | TargetMOI |
| Berberine | Phase 4 | 2086-83-1 | 336.36 |  | TargetMOI |
| Betahistine dihydrochloride | Phase 4 | 5579-84-0 | 209.12 | T0246 | TargetMOI |
| Buserelin acetate | Phase 4 | 68630-75-1 | 1299.48 | TP1307 | TargetMOI |
| Catechin | Phase 4 | 154-23-4 | 290.27 | T0822 | TargetMOI |
| Chidamide | Phase 4 | 1616493-44-7 | 390.41 | T4481 | TargetMOI |
| Curcumin | Phase 4 | 458-37-7 | 368.39 | T1516 | TargetMOI |
| Cyclic somatostatin Acetate(38916-34-6(free base)) | Phase 4 | TP1035L | 1637.88 | TP1035L | TargetMOI |
| Doxifluridine | Phase 4 | 3094-09-5 | 246.19 | T1600 | TargetMOI |
| Embelin | Phase 4 | 550-24-3 | 294.39 | T6485 | TargetMOI |
| (E/Z)-Endoxifen | Phase 4 | 110025-28-0 | 373.5 | T4281 | TargetMOI |
| FG-4592 | Phase 4 | 808118-40-3 | 352.34 | T2515 | TargetMOI |
| Genistein | Phase 4 | 446-72-0 | 270.24 | T1737 | TargetMOI |
| Lobaplatin | Phase 4 | 135558-11-1 | 397.33 |  | Hölzel |
| MK-0752 | Phase 4 | 471905-41-6 | 442.9 | T2625 | TargetMOI |
| Nedaplatin | Phase 4 | 95734-82-0 | 303.18 | T2410 | TargetMOI |
| Perifosine | Phase 4 | 157716-52-4 | 461.66 | T2492 | TargetMOI |
| Quercetin | Phase 4 | 117-39-5 | 302.24 | T2174 | TargetMOI |
| Rapamycin | Phase 4 | 53123-88-9 | 914.18 | T1537 | TargetMOI |
| Silibinin | Phase 4 | 22888-70-6 | 482.44 | T1660 | TargetMOI |
| Quizartinib | Phase 3 | 950769-58-1 | 560.67 | T2066 | TargetMOI |
| Masitinib | Phase 3 | 790299-79-5 | 498.64 | T2609 | TargetMOI |
| Atrasentan | Phase 3 | 173937-91-2 | 510.62 | TQ0259 | TargetMOI |
| Nimorazole | Phase 3 | 6506-37-2 | 226.23 | T2046 | TargetMOI |
| Pyrotinib | Phase 3 | 1269662-73-8 | 583.08 | T7818 | TargetMOI |
| Zosuquidar trihydrochloride | Phase 3 | 167465-36-3 | 636.99 | T6018 | TargetMOI |
| Zibotentan | Phase 3 | 186497-07-4 | 424.43 | T6258 | TargetMOI |
| Veliparib | Phase 3 | 912445-05-7 | 317.21 | T2105 | TargetMOI |
| Vatalanib | Phase 3 | 212141-51-0 | 419.73 | T6720 | TargetMOI |
| TSU-68 | Phase 3 | 252916-29-3 | 310.35 | T6184 | TargetMOI |
| Tivantinib | Phase 3 | 905854-02-6 | 369.42 | T6117 | TargetMOI |
| Tariquidar | Phase 3 | 206873-63-4 | 646.73 | T6287 | TargetMOI |
| Rigosertib sodium | Phase 3 | 592542-60-4 | 473.47 | T5818 | TargetMOI |
| Radotinib | Phase 3 | 926037-48-1 | 530.5 |  | TargetMOI |
| Palifosfamide | Phase 3 | 31645-39-3 | 221.02 | T4350 | TargetMOI |
| Obatoclax | Phase 3 | 803712-79-0 | 413.49 | T6275 | TargetMOI |
| Motesanib | Phase 3 | 453562-69-1 | 373.46 | T2288 | TargetMOI |
| Entinostat | Phase 3 | 209783-80-2 | 376.41 | T6233 | TargetMOI |
| Cediranib | Phase 3 | 288383-20-0 | 450.51 | T2500 | TargetMOI |
| Anacetrapib | Phase 3 | 875446-37-0 | 637.51 | T1928 | TargetMOI |
| Alisertib | Phase 3 | 1028486-01-2 | 518.92 | T2241 | TargetMOI |

**Table S2: Summary of the 36 BM patients from whom establishment of BM-derived spheroid cultures was attempted.**

| **Case ID** | **Gender** | **Age at the diagnosis with BM (years)** | **Primary tumor diagnosis** | **Success of *in vitro* culture establishment** |
| --- | --- | --- | --- | --- |
| BM01 | f | 60 | NSCLC | yes |
| BM02 | f | 73 | Breast carcinoma | no |
| BM03 | f | 66 | NSCLC | yes |
| BM04 | m | 47 | NSCLC | yes |
| BM05 | m | 65 | SCLC | yes |
| BM06 | f | 65 | NSCLC | yes |
| BM07 | m | 75 | Melanoma | yes |
| BM08 | f | 54 | NSCLC | no |
| BM09 | m | 55 | ESCA | yes |
| BM10 | m | 64 | ESCA | no |
| BM11 | m | 55 | NSCLC | no |
| BM12 | f | 51 | SCLC | no |
| BM13 | m | 52 | Melanoma | yes |
| BM14 | f | 78 | NSCLC | yes |
| BM15 | m | 56 | NSCLC | yes |
| BM16 | m | 69 | SCLC | yes |
| BM17 | f | 66 | NSCLC | yes |
| BM18 | f | 83 | NSCLC | yes |
| BM19 | m | 65 | Bladder carcinoma | no |
| BM20 | m | 86 | NSCLC | yes |
| BM21 | f | 71 | NSCLC | yes |
| BM22 | f | 46 | Breast carcinoma | yes |
| BM23 | m | 63 | NSCLC | no |
| BM24 | m | 63 | NSCLC | no |
| BM25 | m | 70 | ESCA | yes |
| BM26 | f | 78 | NSCLC | yes |
| BM27 | f | 85 | Bladder carcinoma | yes |
| BM28 | f | 47 | NSCLC | yes |
| BM29 | m | 48 | Melanoma | yes |
| BM30 | m | 69 | NSCLC | yes |
| BM31 | f | 66 | Melanoma | yes |
| BM32 | m | 57 | GC | yes |
| BM33 | f | 57 | Breast carcinoma | no |
| BM34 | m | 57 | NSCLC | no |
| BM35 | f | 58 | NSCLC | yes |
| BM36 | m | 59 | ESCA | yes |

Abbreviations: NSCLC, non-small cell lung cancer; GC, gastric carcinoma, ESCA: esophageal carcinoma

| **Case ID** | **Target genes carrying genetic alterations** | **Therapeutic compounds and their targets** |
| --- | --- | --- |
| BM28 | *STK11/TP53* | Ultixertinib/ERK inhibitor [1]  Entinostat, Tucidinostat, Vorinostat, CI994/HDAC inhibitors [2]  Tanespimycin/HSP90 inhibitor [3] |
| BM31 | *KIT/JAK3* | Fedratinib, Pacritinib/JAK inhibitors [4, 5] |
| BM32 | *ATM* | Cobimetinib, Trametinib/MEK inhitors [6]  Entinostat, Panobinostat, Vorinostat/HDAC inhibitors [2]  Tanespimycin/HSP90 inhibitor [3] |
| BM35 | *FGFR3 /KRAS* | Vandetanib, Brigatinib, Nintedanib/FGFR&EGFR inhibitors [7-9] |
| BM36 | *JAK3* | Pacritinib, Quizartinib, Momelotinib, Fedratinib [4, 5] |

**Table S3: Therapeutic compounds identified by high-throughput drug screening that targeted BM-specific individual gene alterations.**

**
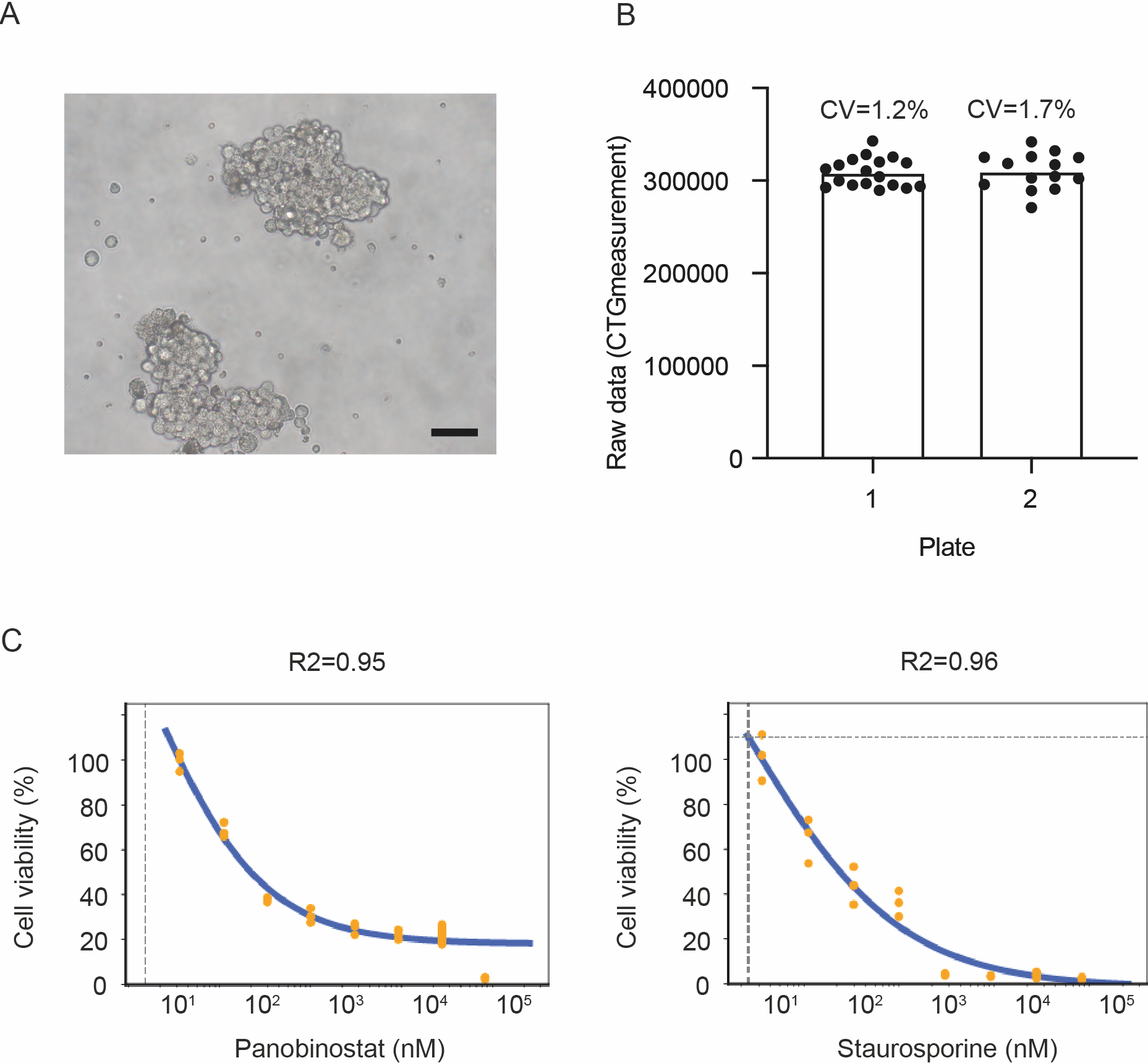
**

**Fig. S1. Primary cancer cells and quality control in high-throughput drug screening (HTS).** A) Representative picture of primary cancer cell neurospheres from BM36 (scale bar = 100 μm). B) To ensure the quality of screening data, each plate typically contains several DMSO controls. It is important to emphasize that this plot is generated using raw viability data measured with CellTiterGlo assay (CTG, Promega). The mean cell viability value of DMSO-treated cell controls on each plate must be similar and the coefficient of variation (CV) lower than 15%. The results were obtained from a HTS assay conducted on BM36. C) Panobinostat and staurosporine were used as proof-of-principle compounds. A typical sigmoidal dose-response curve is presented. R2 must be greater than 0.8. The results were obtained from a HTS assay conducted on BM36.


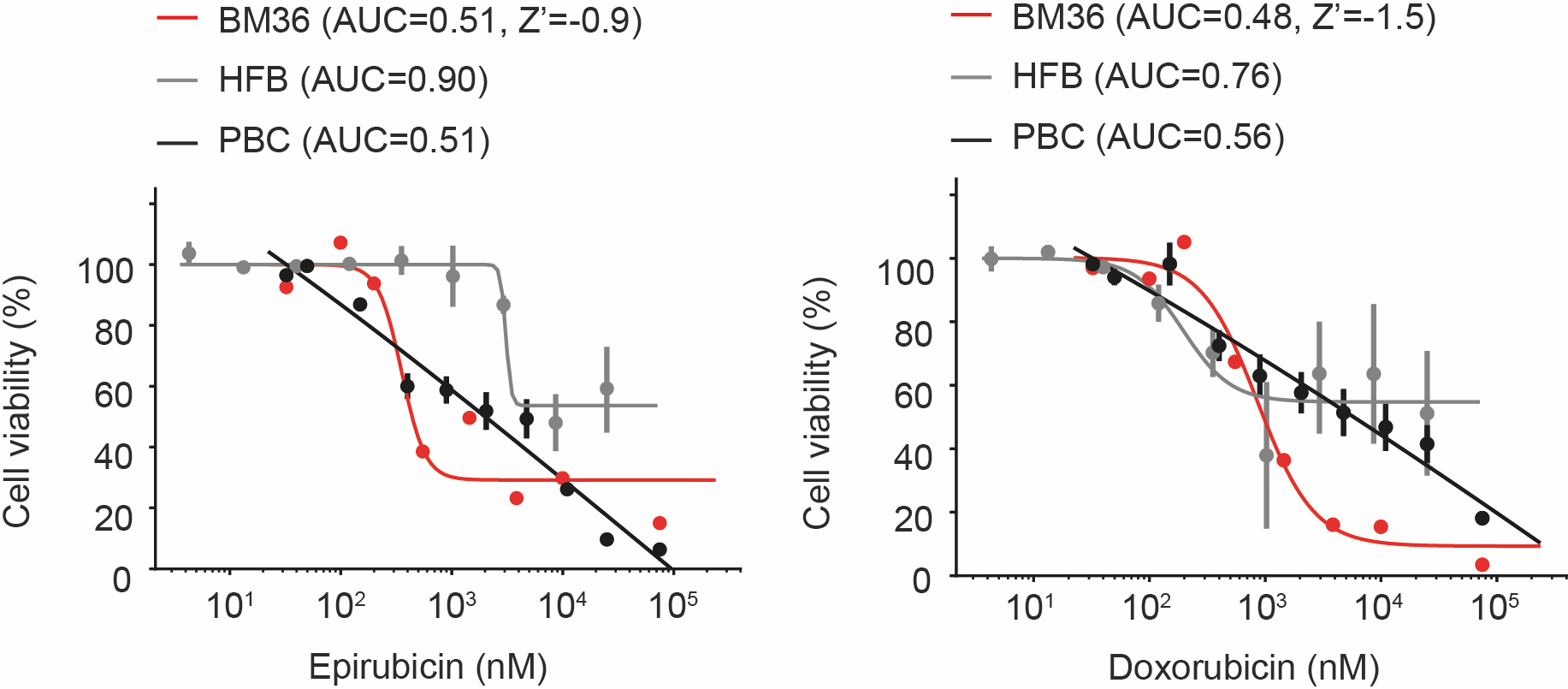


**Fig. S2. Dose-response curves of selected drugs.** Z’ of primary cancer cells is bigger than -2. This indicates that epirubicin (left) and doxorubicin (right) are highly toxic to healthy cells and suppress the growth of primary cancer cells and healthy controls. Cell viability was measured with CellTiterGlo.


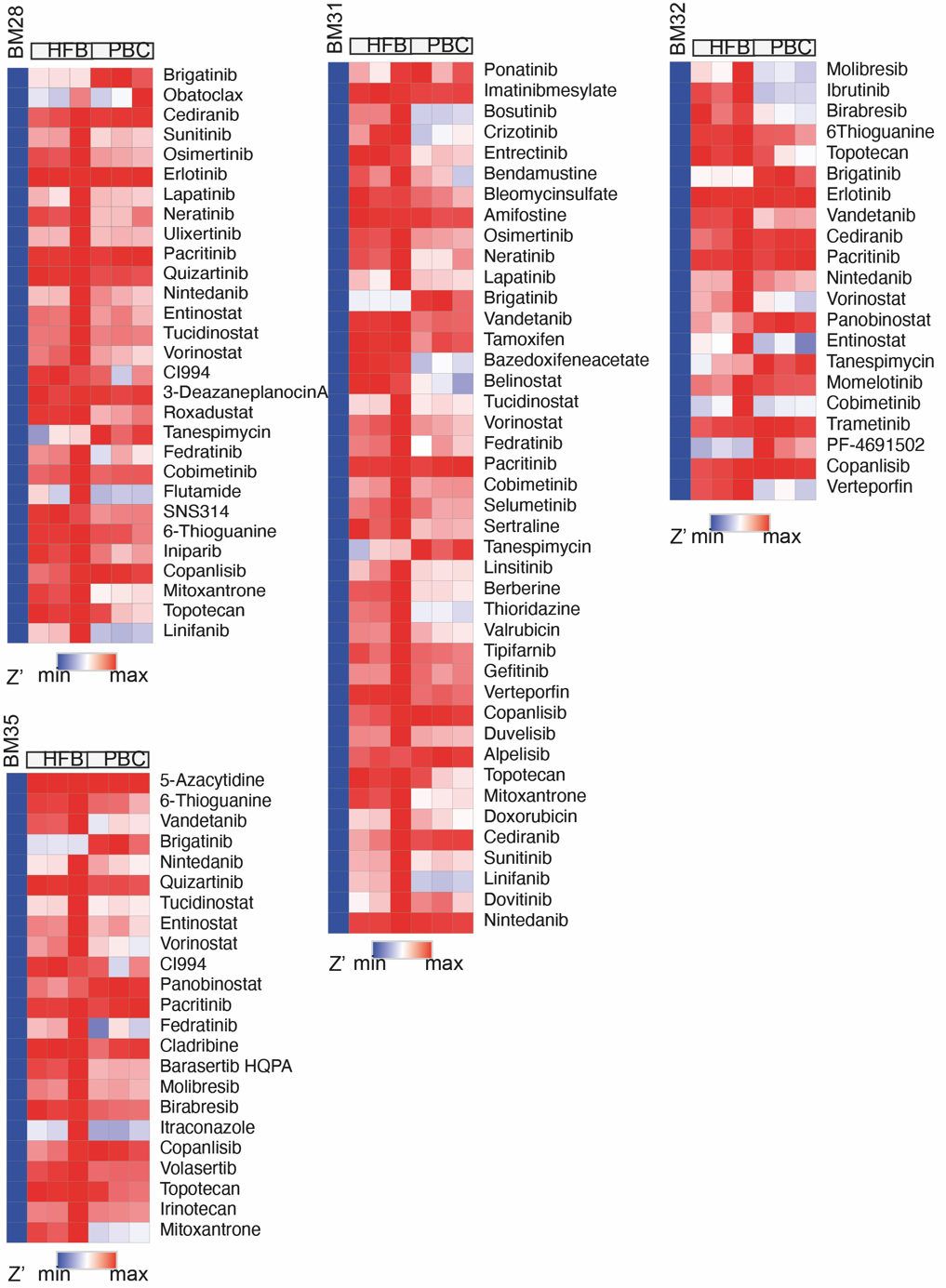


**Fig. S3. Heatmaps of Z’. Drugs with Z’<-2 were presented.**

**References**

1. Caiola E, Iezzi A, Tomanelli M, Bonaldi E, Scagliotti A, Colombo M, Guffanti F, Micotti E, Garassino MC, Minoli L, Scanziani E, Broggini M, Marabese M (2020) LKB1 Deficiency Renders NSCLC Cells Sensitive to ERK Inhibitors. J Thorac Oncol 15: 360-370 doi:10.1016/j.jtho.2019.10.009

2. Juan LJ, Shia WJ, Chen MH, Yang WM, Seto E, Lin YS, Wu CW (2000) Histone deacetylases specifically down-regulate p53-dependent gene activation. J Biol Chem 275: 20436-20443 doi:10.1074/jbc.M000202200

3. Waza M, Adachi H, Katsuno M, Minamiyama M, Tanaka F, Sobue G (2006) Alleviating neurodegeneration by an anticancer agent: an Hsp90 inhibitor (17-AAG). Ann N Y Acad Sci 1086: 21-34 doi:10.1196/annals.1377.012

4. Mullally A, Hood J, Harrison C, Mesa R (2020) Fedratinib in myelofibrosis. Blood Adv 4: 1792-1800 doi:10.1182/bloodadvances.2019000954

5. Regenbogen T, Chen L, Trinkaus K, Wang-Gillam A, Tan BR, Amin M, Pedersen KS, Park H, Suresh R, Lim KH, Ratchford E, Brown A, Lockhart AC (2017) Pacritinib to inhibit JAK/STAT signaling in refractory metastatic colon and rectal cancer. J Gastrointest Oncol 8: 985-989 doi:10.21037/jgo.2017.08.16

6. Smida M, Fece de la Cruz F, Kerzendorfer C, Uras IZ, Mair B, Mazouzi A, Suchankova T, Konopka T, Katz AM, Paz K, Nagy-Bojarszky K, Muellner MK, Bago-Horvath Z, Haura EB, Loizou JI, Nijman SM (2016) MEK inhibitors block growth of lung tumours with mutations in ataxia-telangiectasia mutated. Nat Commun 7: 13701 doi:10.1038/ncomms13701

7. Yang Y, Xu H, Ma L, Yang L, Yang G, Zhang S, Ai X, Zhang S, Wang Y (2021) Possibility of brigatinib-based therapy, or chemotherapy plus anti-angiogenic treatment after resistance of osimertinib harboring EGFR T790M-cis-C797S mutations in lung adenocarcinoma patients. Cancer Med 10: 8328-8337 doi:10.1002/cam4.4336

8. Flaherty KR, Wells AU, Cottin V, Devaraj A, Walsh SLF, Inoue Y, Richeldi L, Kolb M, Tetzlaff K, Stowasser S, Coeck C, Clerisme-Beaty E, Rosenstock B, Quaresma M, Haeufel T, Goeldner RG, Schlenker-Herceg R, Brown KK, Investigators IT (2019) Nintedanib in Progressive Fibrosing Interstitial Lung Diseases. N Engl J Med 381: 1718-1727 doi:10.1056/NEJMoa1908681

9. Brave SR, Odedra R, James NH, Smith NR, Marshall GB, Acheson KL, Baker D, Howard Z, Jackson L, Ratcliffe K, Wainwright A, Lovick SC, Hickinson DM, Wilkinson RW, Barry ST, Speake G, Ryan AJ (2011) Vandetanib inhibits both VEGFR-2 and EGFR signalling at clinically relevant drug levels in preclinical models of human cancer. Int J Oncol 39: 271-278 doi:10.3892/ijo.2011.1022
